# First report of molecular diagnosis of Tunisian hemophiliacs A: Identification of 8 novel causative mutations

**DOI:** 10.1186/1746-1596-7-93

**Published:** 2012-08-10

**Authors:** Hejer Elmahmoudi, Houssein Khodjet-el-khil, Edvard Wigren, Asma Jlizi, Kaouther Zahra, Dorothé Pellechia, Christine Vinciguerra, Balkis Meddeb, Amel Ben Ammar Elggaaied, Emna Gouider

**Affiliations:** 1 Laboratory of Genetics, Immunology and Human Pathologies, Tunis, Tunisia; 2 Department of Medical Biochemistry and Biophysics, Karolinska Institutet, Stockholm, Sweden; 3 Hemophilia Treatment Center, Aziza Othmana Hospital, Tunis, Tunisia; 4 Biological hematology department EAM 4174, Hospices Civils de Lyon Université de Lyon, Lyon, France

**Keywords:** Hemophilia A, Mutations, Intron 22 inversion, Intron 1 inversion, Inhibitors, Molecular analysis, Tunisia

## Abstract

**Introduction:**

Hemophilia A is an X linked recessive hemorrhagic disorder caused by mutations in the *F8* gene that lead to qualitative and/or quantitative deficiencies of coagulation factor VIII (FVIII). Molecular diagnosis of hemophilia A is challenging because of the high number of different causative mutations that are distributed throughout the large *F8* gene. Molecular studies of these mutations are essential in order to reinforce our understanding of their pathogenic effect responsible for the disorder.

**Aim:**

In this study we have performed molecular analysis of 28 Tunisian hemophilia A patients and analyzed the *F8* mutation spectrum.

**Methods:**

We screened the presence of intron 22 and intron 1 inversion in severe hemophilia A patients by southern blotting and polymerase chain reaction (PCR). Detection of point mutations was performed by dHPLC/sequencing of the coding *F8* gene region. We predict the potential functional consequences of novel missense mutations with bioinformatics approaches and mapping of their spatial positions on the available FVIII 3D structure.

**Results:**

We identified 23 different mutations in 28 Tunisian hemophilia A patients belonging to 22 unrelated families. The identified mutations included 5 intron 22 inversions, 7 insertions, 4 deletions and 7 substitutions. In total 18 point mutations were identified, of which 9 are located in exon 14, the most mutated exonic sequence in the *F8* gene. Among the 23 mutations, 8 are novel and not deposited in the HAMSTeRS database nor described in recently published articles.

**Conclusion:**

The mutation spectrum of Tunisian hemophilia A patients is heterogeneous with the presence of some characteristic features.

**Virtual slides:**

The virtual slide(s) for this article can be found here:

http://www.diagnosticpathology.diagnomx.eu/vs/1693269827490715

## Introduction

Hemophilia A is an X linked hereditary bleeding disorder which results from a deficiency or abnormality in FVIII activity. The FVIII protein is composed of structural domains represented as A1-A2-B-A3-C1-C2 and is activated after the dissociation of the B domain. A large and heterogeneous spectrum of mutations has been identified in the *F8* gene [1]. They include partial or complete gene deletions, duplications, large insertions, splice alteration, frameshifts as well as nonsense and missense mutations (FVIII mutation database: http://hadb.org.uk). The most common mutations in severe hemophilia A are the intron 22 inversion mutations which occur in 45-50% of severe hemophilia A patients and the intron 1 inversion mutation which has been reported to be present in approximately 5% of patients with severe phenotypes [2,3]. The development of inhibitors in patients presents a major complication of treatment with FVIII, especially in patients with severe forms of hemophilia A [4]. The Hemophilia treatment centre of Aziza Othmana Hospital in Tunisia follows 143 hemophilia A patients. 72 of these patients have been diagnosed with severe, 49 with moderate and 22 with mild hemophilia A.

In a previous study we have determined the haplotype frequency in Tunisian hemophiliacs A concerning only single nucleotide polymorphisms (SNPs) [5]. Our aim in this study has been to identify the molecular genetics of hemophilia A patients for the first time in Tunisia. The characterization, molecular analysis and spectrum of these genetic alterations are reported in this paper.

## Patients and methods

### Patients

**Figure 1:**
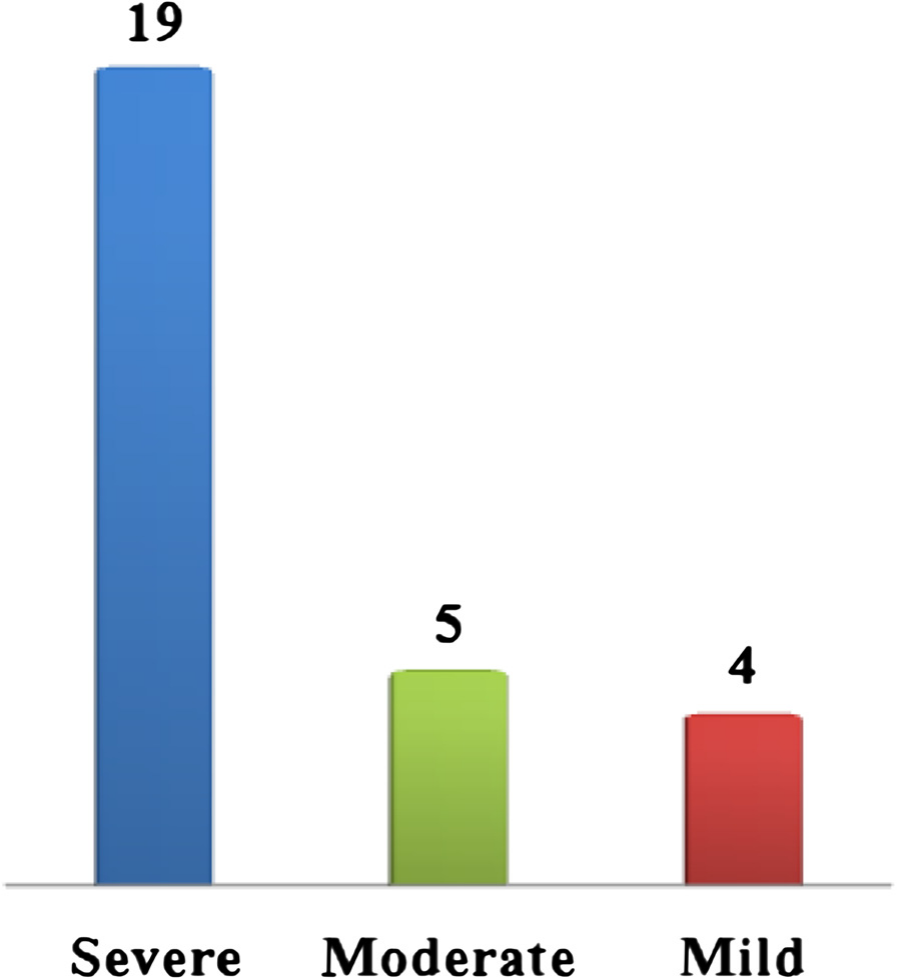
Distribution of Tunisian hemophiliacs A according to their severity.

28 patients with hemophilia A from 22 unrelated families were included in this study. At the time of the study their age ranged between 4 to 38 years. 19 patients had severe form, 5 had moderate form and 4 had mild form of hemophilia A (Figure 1). All the patients gave informed consent for molecular studies.

### Molecular genetic analysis

DNA was extracted from whole blood samples using a phenol chloroform protocol. Severe hemophilia A patients were first screened for intron 22 inversion. Negative patients were then tested for intron 1 inversion. Intron 22 inversion was detected using LD PCR and confirmed with southern blot as described [6]. Intron 1 inversion was performed using the Bagnall’s protocol [7].

PCR amplification was performed for mild, moderate and severe hemophilia A patients without intron 22 or intron 1 inversions. The coding sequence of *F8* gene was divided into 33 amplicons (200-480 bp). Exon 14 was divided into 8 fragments for PCR amplification and the other exons were amplified as unique fragments. Primer sequences, annealing temperatures and the size of PCR fragments are available from the corresponding author, on request. Mixtures of patients and wild type DNA were screened by denaturing high liquid performance (dHPLC) on a WAVE DNA Fragment Analysis System (Transgenomics, San Jose, USA). All large deletions were defined as a consistent absence of PCR amplification products. Altered profile revealed by dHPLC were then sequenced in both strands using ABI Dye Terminator Cycle Sequencing (Perkin-Elmer Applied Biosystems, Foster City, CA, USA) and analyzed using a capillary sequencer Genetic Analyser ABI PRISM310 (Perkin-Elmer Applied Biosystems, Foster City, CA, USA). Mutations were confirmed by a second PCR and sequencing. Results were analyzed using BLAST (http://www.ncbi.nlm. nih.gov/blast) program in comparison with the wild-type *F8* gene sequence. Mutation nomenclature was given according to HAMSTeRS as well as in parentheses according to the international recommendations for the description of sequence variants on the Human Genome Variation Society (HGVS) website (http://www.hgvs.org).

### Analysis of missense mutations

All identified *F8* mutations were compared to those described in the HAMSTeRS database (http://www.HAMSTeRS.ac.uk/). PolyPhen software (Polymorphism Phenotyping) was used to perform the sequence alignment of the homologous FVIII from four mammalian species (human, murine, canine and pig) and to predict the possible impact of an amino acid substitution on the structure and function of FVIII. The 3D structure of FVIII was visualized using the PyMOL Molecular Graphics System (http://www.pymol.org) [8].

## Result

**Figure 2:**
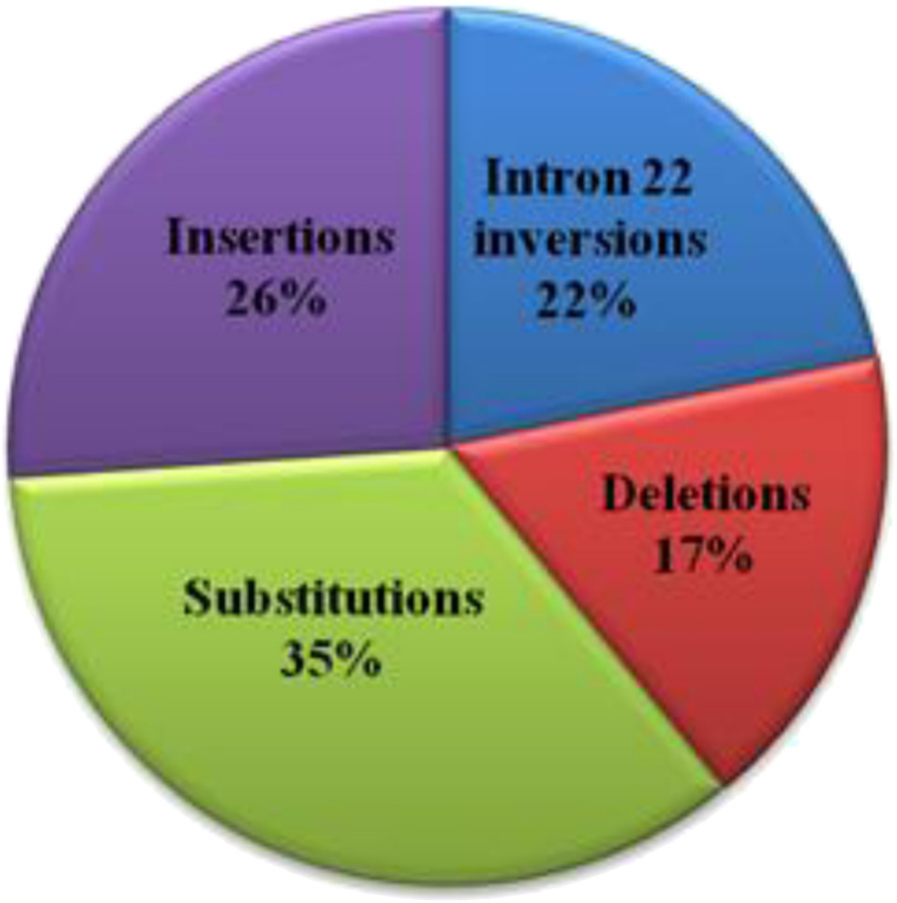
Frequencies of identified mutations according to their types.

In this study, 28 hemophilia A patients from 22 unrelated families were investigated. In total we identified 23 different mutations, which included 5 intron 22 inversions, 8 substitutions, 6 insertions and 4 deletions identified in 4 severe hemophilia A patients (Figure 2, Tables 1 and 2). Among the 23 different mutations, 8 are novel mutations and have not been described in HAMSTeRS database or reported in recently published data. Of these novel mutations, 6 occur in severe hemophilia A patients (del exons 1–13, intron 22 inversion (18 kb, 15.5 kb and 14 kb), p.1595(1614)DfsX40, p.12(31)LfsX11, p.C179(198)R and p.N784(803)N) and 2 occur in moderate hemophilia A patients (p.727(746)SfsX7 and p.G520(539)R).

**Table 1:** Identified types of intron 22 inversion in severe Tunisian hemophiliacs A

**Family**	**Number of Patient**	**FVIII :C**	**Intron 22 Inversion**		
			**Type I**	**Type II**	**Other Type**
Family 8	1	<1%			Yes : 18 kb, 15.5 kb and 14 kb
Family 9	2	<1%		Yes	
Family 10	1	<1%	Yes		
Family 11	2	<1%	yes		
Family 12	1	<1%	yes		

**Table 2:** Identified causative mutations in Tunisian hemophiliacs A

**Family**	**Number of Patient**	**FVIII:C**	**Severity**	**Mutation**	**Type**	**Location**	**Domain**	**Inhibitors**	**Reported**
Family 1	1	<1%	Severe	c.90-91insA; p.12(31)LfsX11	Insertion	Exon 1	A1	No	Novel
Family 2	1	5%	Moderate	c.1615 G>C; p.G520(539)R	Missense	Exon 11	A2	No	Novel
Family 3	1	2.5%	Moderate	ag/GGC>ac/GGC	Splicing	Intron14	B	No	Reported
Family 4	1	<1%	Severe	Exons1-13del	Large deletion	Exons 1-13	A1-A2	Yes	Novel
Family 5	1	<1%	Severe	c.592 T>C; p.C179(198)R	Missense	Exon 4	A1	No	Novel
Family 6	2	<1%	Severe	c.4844ins264pb	Large insertion	Exon 14	A1	No	Novel
Family 7	1	12.5%	Moderate	c.2236-2237insT; p.727(746)SfsX7	Insertion	Exon 14	B	No	Novel
Family 11	2	<1%	Severe	c.3637-3638insA; p.1191(1210)IfsX29	Insertion	Exon 14	B	No	Reported
Family 13	1	<1%	Severe	c.4379-4380insA; p.1441(1460)KfsX2	Insertion	Exon 14	B	No	Reported
Family 14	1	<1%	Severe	c.6873-6876delTC; p.2272(2291)TfsX	Deletion	Exon 25	C2	No	Reported
Family 15	1	<1%	Severe	c.3637-3638delA; p.1191(1210)IfsX5	Deletion	Exon 14	B	No	Reported
Family 16	1	<1%	Severe	c.5071-5075delATGAA; p.1671-3(1690–3)fsX	Deletion	Exon 14	B	No	Reported
Family 17	2	<1%	Severe	c.77 T>C; p.L7(26)P	Missense	Exon 1	A1	No	Reported
Family 18	1	4.4%	Moderate	c.1492 G>A; p.G479(498)R	Missense	Exon 10	A2	No	Reported
Family 19	1	6.5%	Mild	c.1696 C>T; p.L547(566)F	Missense	Exon 11	A2	No	Reported
Family 20	1	1.6%	Moderate	c.3870-3871insA; p.1271(1290)KfsX29	Insertion	Exon 14	B	No	Reported
Family 21	3	12-20%	Mild	c.2167 G>A; p. A704(723)T	Missense	Exon 14	B	No	Reported
Family 22	1	<1%	Severe	c.2409 T>C; p.N784(803)N	Missense	Exon 14	B	No	Novel

**Figure 3:**
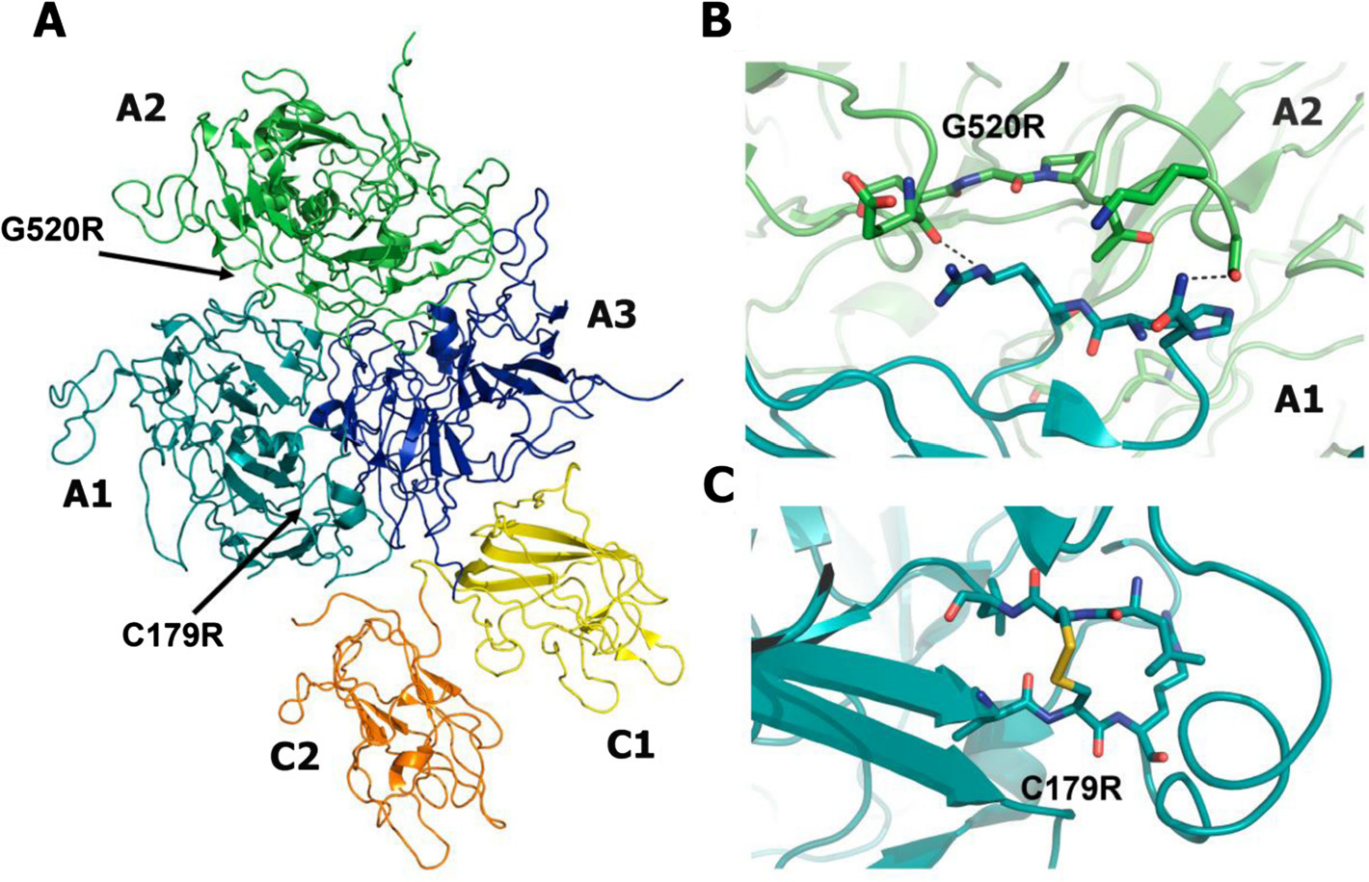
**(A) Crystal structure of B domain-deleted factor VIII (PDB ID 2R7E).** The domains are individually labeled and in different colors. The positions of the mutations studied in this article are indicated by arrows. Close-up views of the positions of the mutations with the mutated and nearby residues shown as stick models and hydrogen bonds as dashed lines. (**B**) The p.G520(539)R mutation located in the interface between the A1 and A2 domains and (**C**) the p.C179(198)R mutation located in A2 domain.

Analysis of the two novel missense mutations p.C179(198)R and p.G520(539)R using the PolyPhen Software suggested both mutations to be considered as structurally damaging. Further analysis, mapping the substitution sites on the 3D structure of FVIII, show that both of these mutated residues are involved in interaction that can be considered important for the structural integrity of the mature FVIII protein. Residue C179(198) forms a disulfide bond with the residue C153. The mutation p.C179(198)R disrupts this disulfide bond formation, and thereby most likely leads to destabilization of the A1 domain (Figure 3). The second novel mutation p.G520(539)R is located in the interface between the A1 and A2 domains. Substitution of this neutral glycine with a charged arginine probably disturbs the interactions in this interface and destabilizes the interaction between the A1 and A2 domains (Figure 3).

## Discussion

We report the first molecular study performed in Tunisian hemophilia A patients belonging to Hemophilia Treatment Center of Aziza Othman Hospital. Our cohort consists of 28 patients from 22 unrelated families. Despite the limited number of our cohort, the novel mutations occur with a significant frequency (34%) indicating that our patients have a specific mutational profile in comparison with previously reported data. Each studied family was found to have its unique mutation except for one. Among the 18 identified point mutations 9 were located in exon 14, the largest exonic coding sequence of the *F8* gene. The correlation between genotype/phenotype was assessed for the identified mutations in order to clarify the effect of mutations on the phenotype and to compare our data with previously reported data.

### Severe hemophilia A patients

#### Inversions (5)

In the 19 patients with severe hemophilia A, intron 22 inversion was identified in 7 patients (36.84%) belonging to five different families, while no family present intron 1 inversion (0%). This result indicates that the intron 22 inversion, which is reported in severe hemophilia A in the general population with a frequency of 45%-50% [9], is less frequent in our patient cohort. The same tendency was found for the intron 1 inversion, which is reported with a frequency of 5% [10]. When we compare our findings with previous data from other Arab countries the frequencies of these 2 inversions are lower in our patients [11-14]. This result shows that our patients have a specific genetic profile which is confirmed by other molecular studies concerning our country [15]. Nevertheless, this data must be confirmed with a larger number of studied patients.

Among the identified intron 22 inversion types, the type I inversion was the most frequent in our patients (families 10, 11 and 12), which correlates with what previously has been reported in the literature [16]. The type II inversion was identified in one patient (family 9) and a novel atypical pattern (family 8) with 3 bands (18 kb, 15.5 kb and 14 kb), since the amplification of exons 22 and 23 gave the expected size this atypical pattern may be explained by a deletion of 2 kb in the INT22-h1 or in the INT22-h3 homologous regions (Table 1).

#### Insertions and deletions (8)

In our cohort of severe patients we identified 7 frameshift mutations (Table 2). All of them are associated with very low or undetectable FVIII:C in plasma leading to a severe phenotype as expected, since frameshift mutations resulting from insertions or/deletions in most cases lead to severe phenotypes [17]. The new large deletion spanning exons 1-13 was found in one patient with severe hemophilia A and was associated with development of inhibitors (family 4).

#### Substitutions (3)

We identified 3 substitutions in 3 unrelated families with severe hemophilia A (Table 2), among these the novel mutation p.C179(198)R, which occurs in exon 4 within the A1 domain (family 5). In the encoded protein this mutation substitutes a conserved cystein residue and disrupts the disulfide bond formation leading to destabilization of the A1 domain. The severe phenotype associated with this mutation is in accordance with previously reported genotype/phenotype correlation. For instance, the mutation p.C179(189)G, which is also associated with a severe phenotype, is reported twice in HAMSTeRS [18]. Previous studies have also highlighted that mutations of conserved amino acids in FVIII frequently are associated with a severe phenotype [19,20].

The second novel mutation, p.N784(803)N, is a silent mutation identified in one patient with severe hemophilia A (family 22). This silent mutation has no effect on the mature FVIII protein structure, but it might play a role in the expression levels of the protein, such as other silent mutations which have been recently identified [21]. To confirm this hypothesis we need to screen this point mutation in healthy individuals in order to exclude that it is not associated with a SNP, since it is not reported in the HAMSTeRS data base or in recent identified SNPs [22]. The exact effect of this mutation remains elusive and will be of interest for future studies to reveal. The finding that the mutation p.L7(26)P identified in family 17 is associated with a severe phenotype is in accordance with previous findings, which have shown that the same mutation causes a severe phenotype [23].

#### Inhibitor development

The type and location of the *F8* gene mutation is an important determinant of inhibitor development in patients with severe hemophilia A. In a recent study, nonsense mutations and large deletions were shown to be associated with a higher risk of inhibitor development than intron 22 inversion, small deletions/insertions and missense mutations [24]. In our cohort no nonsense mutation was identified and only one patient with a large deletion spanning the exons 1–13 developed inhibitors. None of the patients with insertions/small deletions or missense mutations developed inhibitors. The low incidence of inhibitor development in our cohort (1/28) and (6/143) in total for all patients followed in Hemophilia Treatment Center [25] may be explained by the low frequency of nonsense mutations and large deletions in our cohort. In order to confirm this hypothesis we need to increase the number of the investigated hemophilia A patients in our study.

### Moderate hemophilia A patients

#### Insertions (2)

The novel insertion c.2236-2237insT and the previously reported insertion c.3870-3871insA identified in family 7 and 20 respectively (Table 2), are due to insertions of a ‘T’ nucleotide in the stretch of 3Ts at codon 727(746) and of an ‘A’ nucleotide in the stretch of 9As at codon1271(1290). Insertions in the stretch of poly A nucleotides in exon 14 of the *F8* gene are the cause of the moderate clinical severity in some cases [26,27]. Accordingly to these reported data, we can consider that also the new insertion of T in the stretches of poly T can be associated with moderate phenotype in our patient.

#### Substitutions and splicing site mutations (3)

The novel p.G520(539)R mutation associated with the moderate phenotype is located within the A1 domain and is part of the A1-A2 domain interface. Reported mutations in HAMSTeRS from position 516(525) to 521(540) located at A1-A2 interface are all associated with moderate or mild phenotypes http://www.HAMSTeRS.ac.uk/, [28]. Studies of residues involved in interaction at the A1-A2 domain interface have showed that reducing the charge and increasing hydrophobicity at this interface yield FVIII with improved stability [29,30]. Thus, the mutation p.G520(539)R, which increases the charge at the A1-A2 interface, most likely has the opposite effect and leads to destabilization of the protein. The reported missense mutation p.G479(488)R, which is associated with a moderate phenotype in our patients, is described in HAMSTeRS with mild moderate or severe form [20]. The splicing site mutations are often associated with a severe phenotype [31]. In contrast, the unique identified splicing mutation (ag/GGC > ac/GGC) in our study, which interferes in the splicing process resulting in the skipping of exon 15, is associated with a moderate phenotype (Table 2).

### Mild hemophilia A patients

#### Substitutions (2)

The mutation p.A704(723)T is reported 9 times in the HAMSTeRS data base, in 5 cases it is associated with the mild form which is in accordance with our result (Table 2). On the other hand the p.L547(566)F has previously been reported in one severe patient while in our study it is associated with a mild form (Table 2). The discrepancy between mutations predicted to be responsible for severe, mild or moderate forms may be explained by the intervention of other hemostatic factors which can modify the clinical severity of hemophilia [32].

## Conclusion

We report here the first molecular analysis of causative mutations in Tunisian hemophiliac A patients. In total we have identified 23 mutations including 8 novel mutations. In the future we will continue this study on patients from our Hemophilia Treatment Center in order to reinforce our understanding in the molecular defect of hemophilia A in Tunisia and to set up counseling genetic and the prenatal diagnosis for Tunisian families with hemophilia A.

## Competing interests

The authors stated that they had no interests which might be perceived as posing a conflict or bias.

## Authors’ contributions

EH performed the research, analyzed the data and wrote the paper; KH and JA analyzed the data; WE performed protein structure analysis and contributed in writing the paper; ZK contributed in the data collection; PD and VC performed the research; MB, EBAA and GE designed the research. All authors read and approved the final manuscript.
